# Synthesis and Evaluation of a Dimeric RGD Peptide as a Preliminary Study for Radiotheranostics with Radiohalogens

**DOI:** 10.3390/molecules26206107

**Published:** 2021-10-10

**Authors:** Hiroaki Echigo, Kenji Mishiro, Takeshi Fuchigami, Kazuhiro Shiba, Seigo Kinuya, Kazuma Ogawa

**Affiliations:** 1 Graduate School of Medical Sciences, Kanazawa University, Kanazawa 920-1192, Japan; h.echigo1010@gmail.com (H.E.); t-fuchi@p.kanazawa-u.ac.jp (T.F.); 2 Institute for Frontier Science Initiative, Kanazawa University, Kanazawa 920-1192, Japan; mishiro@p.kanazawa-u.ac.jp; 3 Research Center for Experimental Modeling of Human Disease, Kanazawa University, Takara-machi 13-1, Kanazawa 920-8640, Japan; shiba@med.kanazawa-u.ac.jp; 4 Department of Nuclear Medicine, Kanazawa University Hospital, Kanazawa University, Takara-machi 13-1, Kanazawa 920-8641, Japan; kinuya@med.kanazawa-u.ac.jp

**Keywords:** RGD peptide, integrin, tumor, alpha-particle

## Abstract

We recently developed ^125^I- and ^211^At-labeled monomer RGD peptides using a novel radiolabeling method. Both labeled peptides showed high accumulation in the tumor and exhibited similar biodistribution, demonstrating their usefulness for radiotheranostics. This study applied the labeling method to a dimer RGD peptide with the aim of gaining higher accumulation in tumor tissues based on improved affinity with α_v_β_3_ integrin. We synthesized an iodine-introduced dimer RGD peptide, E[c(RGDfK)] (**6**), and an ^125/131^I-labeled dimer RGD peptide, E[c(RGDfK)]{[^125/131^I]c[RGDf(4-I)K]} ([^125/131^I]**6**), and evaluated them as a preliminary step to the synthesis of an ^211^At-labeled dimer RGD peptide. The affinity of **6** for α_v_β_3_ integrin was higher than that of a monomer RGD peptide. In the biodistribution experiment at 4 h postinjection, the accumulation of [^125^I]**6** (4.12 ± 0.42% ID/g) in the tumor was significantly increased compared with that of ^125^I-labeled monomer RGD peptide (2.93 ± 0.08% ID/g). Moreover, the accumulation of [^125^I]**6** in the tumor was greatly inhibited by co-injection of an excess RGD peptide. However, a single injection of [^131^I]**6** (11.1 MBq) did not inhibit tumor growth in tumor-bearing mice. We expect that the labeling method for targeted alpha therapy with ^211^At using a dimer RGD peptide could prove useful in future clinical applications.

## 1. Introduction

“Theranostics” is a term that combines “therapeutics” and “diagnostics” and refers to the use of specific agents or techniques that combine diagnosis and targeted therapy [1]. Theranostics has recently gathered considerable attention in oncology as being a safe and effective method for providing personalized medical treatment using a tailored combination of medications to diagnose and subsequently target tumors [2]. In nuclear medicine, cancer is confirmed via molecular imaging with positron emission tomography (PET) or conventional nuclear medicine imaging, including planar or single-photon emission computed tomography (SPECT) imaging, and subsequent therapy involves the delivery of targeted radionuclide therapy with α-particle or β-particle emitter radionuclides. Supposing the diagnostic and therapeutic radiopharmaceuticals show similar biodistribution, absorbed doses in the tumor and each normal tissue during therapy can be calculated based on the quantitative imaging at the time of diagnosis. As the therapeutic effects and side effects of delivered radionuclides are predictable, we can more accurately select the correct therapeutics for each individual patient prior to treatment. Therefore, nuclear medicine is a reasonable method for integrating theranostics. The method of using radioisotopes for theranostics is called “radiotheranostics” [3,4].

In nuclear medicine therapy, targeted alpha therapy (TAT) has gained much attention because of the excellent therapeutic effects derived from the high linear energy transfer (LET) of alpha-particles [5]. Among various alpha emitters, ^211^At has become more popular because the half-life (t_1/2_ = 7.2 h) of ^211^At could be sufficient for TAT, and ^211^At can be produced from natural bismuth targets via the ^209^Bi(α,2n)^211^At nuclear reaction by cyclotron [6]. Numerous promising preclinical studies with ^211^At have been reported in recent years [7].

We recently developed a novel ^211^At-labeling method for peptides using RGD peptide as a model peptide [8]. The ^211^At-labeled RGD peptide [^211^At]c[RGDf(4-At)K] and the corresponding radioiodine-labeled RGD peptide [^125^I]c[RGDf(4-I)K] showed high accumulation in the tumor and similar biodistribution. The results indicated that radiotheranostics combining [^123^I]c[RGDf(4-I)K] imaging and [^211^At]c[RGDf(4-At)K] therapy is possible. On the other hand, the superior biodistribution of tracers, such as higher tumor accumulation, prolonged tumor retention, and lower uptake in normal tissues, would be required for specific therapeutic effects. As one of the strategies to improve the tracer characteristics, it has been reported that the affinity of RGD peptide for α_v_β_3_ integrin is improved by the usage of multivalent peptides, such as dimeric peptides or tetrameric peptides [9].

**Figure 1:**
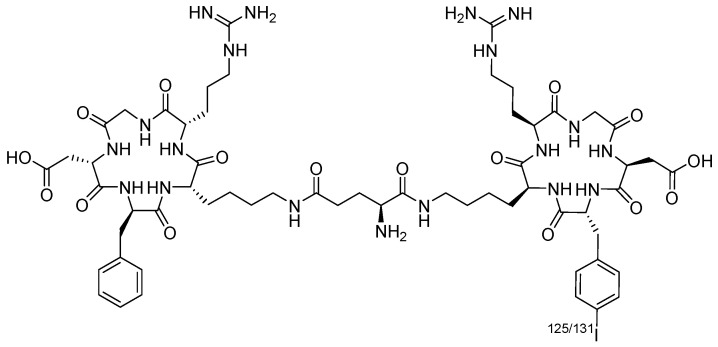
A chemical structure of [^125/131^I]**6**.

In this study, we hypothesize that applying the labeling method to a dimeric RGD peptide leads to the development of superior probes for radiotheranostics using a combination of radioiodine and ^211^At. Thus, we synthesized and evaluated the ^125/131^I-labeled dimer RGD peptide E[c(RGDfK)]{[^125/131^I]c[RGDf(4-I)K]} ([^125/131^I]**6**) (Figure 1) as a preliminary step for radiotheranostics with ^211^At-labeled dimer RGD peptide. This is because the radiolabeling with radiohalogens such as ^125/131^I and ^211^At can be achieved using the same tributyltin precursors, and ^125^I- and ^211^At-labeled probes showed very similar biodistribution patterns in our previous studies [8,10,11]. Moreover, ^125^I is commercially available and has a long half-life (t_1/2_ = 59.4 d), which is appropriate for fundamental research. ^131^I is also commercially available and was used for radionuclide therapy to compare the therapeutic effects of ^211^At in the future.

## 2. Results

### 2.1. Preparation of [^125/131^I]***6***

**Scheme 1:**
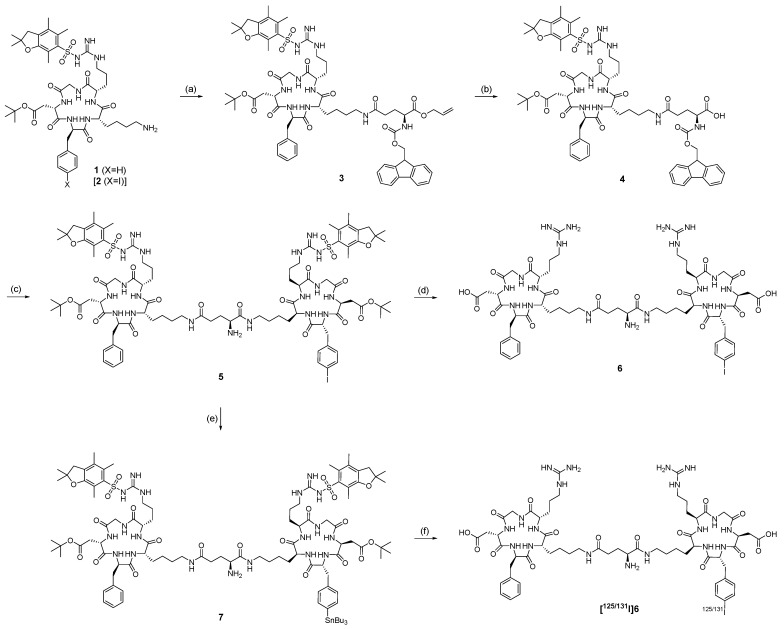
Synthetic scheme of **6** and [^125/131^I]**6**. Reagents: (**a**) Fmoc-Glu-OAll, TBTU, DIPEA, DMF (**b**) phenylsilane, DMF (**c**) (i) c[R(Pbf)GD(O*t*Bu)f(4-I)K] (**2**), TBTU, DIPEA, DMF (ii) piperidine (**d**) TFA, water, TIS. (**e**) Bis(tributyltin), tris(dibenzylideneacetone)dipalladium(0), DIPEA, methanol (**f**) (i) [^125^I]NaI, NCS, acetic acid, acetonitrile (ii) TFA, water, TIS.

Scheme 1 shows a synthetic scheme of [^125/131^I]**6** and its precursor. After **1** and **2** were synthesized using a general Fmoc solid-phase synthesis method, **3** was synthesized by conjugating Fmoc-Glu-OAll with **1**. **4** was synthesized by deprotecting the allyl group of **3**, and **5** was synthesized by conjugation of **4** with **2**. **6** was synthesized by deprotection of **5**. The iodo group in **5** was replaced with a tributylstannyl group via a Pd-catalyzed stannylation reaction to synthesize **7**. We then performed ^125/131^I-labeling and deprotection of the protecting groups. The radiochemical yields in the two-step method of [^125^I]**6** and [^131^I]**6** were 38% and 24%, respectively. Following HPLC purification, [^125^I]**6** and [^131^I]**6** had radiochemical purities of over 96% and 92%, respectively. The total radiosynthesis time for [^125/131^I]**6** was about 3 h including HPLC purification. The radiochemical yields of [^125/131^I]**6** were low due to the complicated radiolabeling procedure by two steps. The identities of [^125/131^I]**6** were verified by comparing their retention times with **6** (Figure S1). Although the radiochemical purity of [^131^I]**6** was not enough even after HPLC purification, the therapeutic experiments were performed without further purification because the amount of radioactivity was prioritized.

### 2.2. α_v_β_3_ Integrin Binding Assay

**Figure 2:**
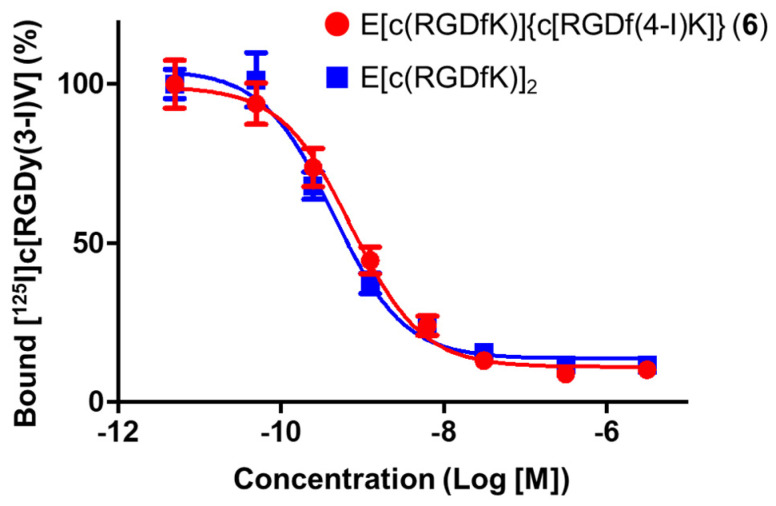
α_v_β_3_ Integrin binding assay. Representative displacement curves of the competitive binding of [^125^I]c[RGDy(3-I)V] to α_v_β_3_ integrin on U-87 MG cells with E[c(RGDfK)]_2_ and **6**. Error bars represent SD.

The affinity of **6** and E[c(RGDfK)]_2_, a dimer RGD peptide without iodine, for α_v_β_3_ integrin was determined via a competitive binding assay with U-87 MG cells. Representative displacement curves of the assay are shown in Figure 2. Binding of the radioligand [^125^I]c[RGDy(3-I)V] to α_v_β_3_ integrin was inhibited by **6** and E[c(RGDfK)]_2_ in a concentration-dependent manner. The half-maximal inhibitory concentration (IC_50_) values (nM) for **6** and E[c(RGDfK)]_2_ were 1.2 ± 0.5 and 0.8 ± 0.4 (mean ± SD for three independent experiments), respectively. The results indicate that **6** and E[c(RGDfK)]_2_ possess a specific affinity for α_v_β_3_ integrin. Furthermore, these similar values of IC_50_ of **6** and E[c(RGDfK)]_2_ indicate that the introduction of an iodine atom into the phenylalanine residue did not significantly impede the affinity of E[c(RGDfK)]_2_ for the α_v_β_3_ integrin.

### 2.3. Determination of the Partition Coefficient

An experimental Log *p* value of [^125^I]**6** was −2.33 ± 0.04. The value was higher than that of the monomeric radioiodine-labeled RGD peptide [^125^I]c[RGDf(4-I)K] (−3.04 ± 0.46) from our previous study [8]. This result indicates that lipophilicity was increased by dimerization of the RGD peptide.

### 2.4. In Vitro Stability

An in vitro stability experiment of [^125^I]**6** in PBS(−) (pH 7.4) solution was performed. After incubation at 37 °C for 24 h, 90.8 ± 0.7% (mean ± SD for three samples) of its radioactivity remained intact.

### 2.5. Biodistribution Experiments

**Figure 3:**
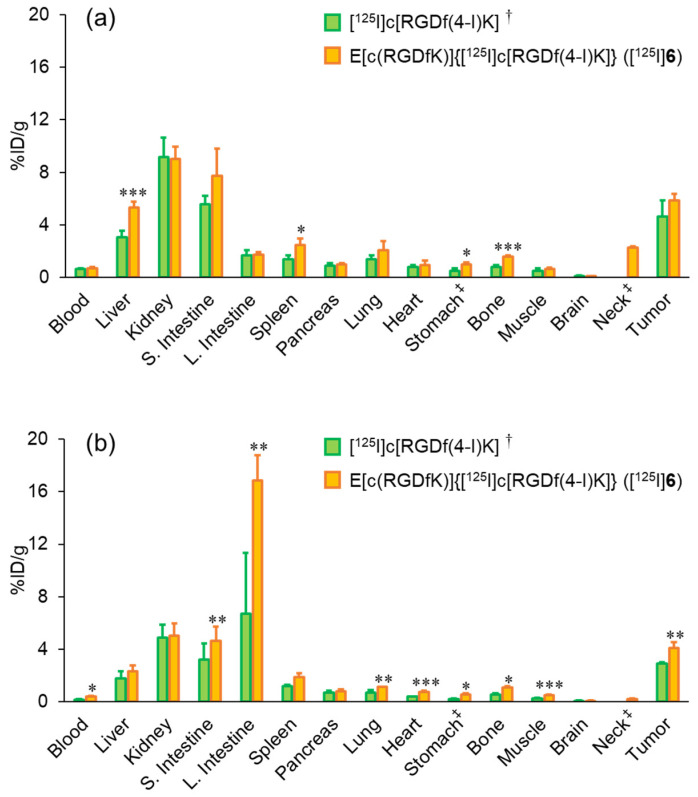
Biodistribution experiments. Biodistribution of radioactivity at (**a**) 1 h and (**b**) 4 h after intravenous injection of [^125^I]c[RGDf(4-I)K]^†^ and [^125^I]**6** in U-87 MG tumor bearing mice (mean ± SD). ^‡^ Expressed as % injected dose. Significance was determined by unpaired Student’s *t*-test (* *p* < 0.05, ** *p* < 0.01, *** *p* < 0.001). ^†^ Data from reference [8]. No data of neck for [^125^I]c[RGDf(4-I)K].

A comparison of the biodistribution experiments in U-87 MG tumor mice between [^125^I]**6** and [^125^I]c[RGDf(4-I)K] is shown in Figure 3 and Table S1. [^125^I]**6** was highly accumulated in the tumor based on the results of the in vitro assay. Specifically, at 4 h postinjection, the tumor accumulation of [^125^I]**6** (4.12 ± 0.42% ID/g) was significantly higher than that of [^125^I]c[RGDf(4-I)K] (2.93 ± 0.08% ID/g) [8]. The accumulation of [^125^I]**6** in the liver and intestines also tended to be higher than that of [^125^I]c[RGDf(4-I)K]. Meanwhile, it is known that the radioactive accumulation in the thyroid gland and stomach is an index of deiodination of radioiodine labeled probes. In this study, the accumulation of radioactivity in the neck containing the thyroid glands and stomach was low (Figure 3), suggesting that in vivo deiodination of [^125^I]**6** hardly occurred.

**Figure 4:**
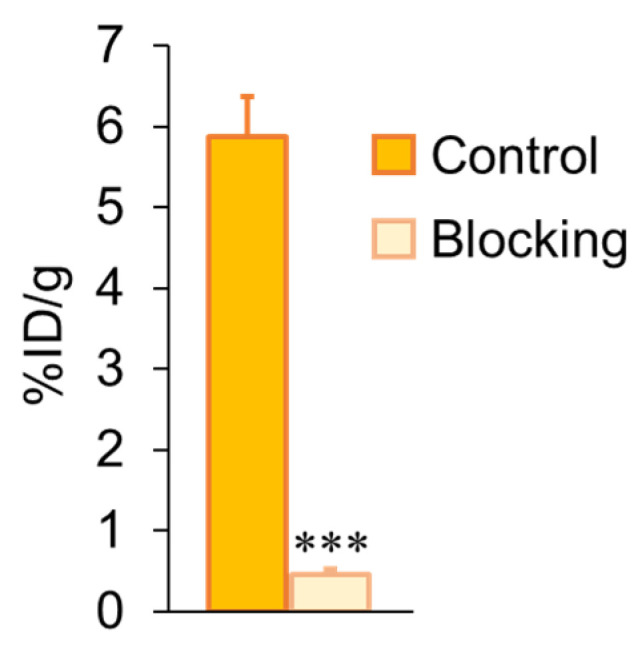
Blocking study. Comparison of tumor uptake (mean ± SD) of [^125^I]**6** at 1 h postinjection under no-carrier-added conditions and with co-injection of an excess of c(RGDfK). Significance was determined by unpaired Student’s *t*-test (*** *p* < 0.001).

An in vivo blocking study was performed to evaluate whether tumor accumulation was derived from α_v_β_3_ integrin specificity. The effect of c(RGDfK) on tumor uptake of [^125^I]**6** at 1 h postinjection is shown in Figure 4. Co-injection of an excess of c(RGDfK) drastically decreased the tumor uptake of [^125^I]**6**, indicating that the tumor accumulation of [^125^I]**6** is caused by its specific binding via α_v_β_3_ integrin. Moreover, it also significantly reduced the accumulation of radioactivity in numerous types of normal tissues (Table S1). It is known that α_v_β_3_ integrin is expressed in the microvessels of normal tissues, such as the liver and lungs [12]. Thus, the result of the blocking study is reasonable for [^125^I]**6** as an α_v_β_3_ integrin-directing agent.

### 2.6. Radionuclide Therapy

**Figure 5:**
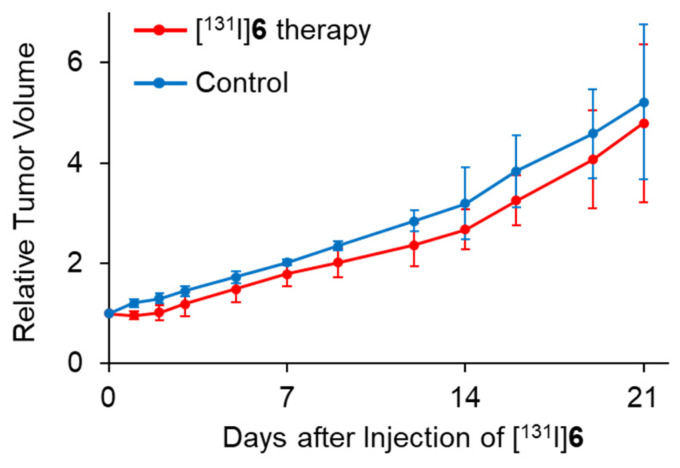
Curves depicting inhibition of growth of U-87 MG on therapy with [^131^I]**6** compared with no treatment. Data are expressed as tumor volume relative to that on day of treatment (mean ± SD).

The tumor volume and body weight of tumor-bearing mice after treatment in the [^131^I]**6** treatment and control groups are shown in Figure 5 and Figure S2, respectively. There was no significant difference in tumor volume and body weight between the treatment and control groups.

## 3. Discussion

We recently reported the simple one-step reaction of an ^125^I- and ^211^At-labeling method of a monomeric RGD peptide via a Pd-catalyzed stannylation reaction after deprotection [8], whereas, in this study, deprotection after the ^125/131^I-labeling reaction, namely the two-step reaction, was performed because the Pd-catalyzed stannylation reaction failed after deprotection. The reason for this failure is not apparent; however, the yield of the stannylation reaction of the monomeric RGD peptide is lower (25%) than that of other stannylation reactions in previous studies (45–75%) [8,13,14]. We suppose that this difference might be derived from impeding the efficient stannylation reaction by functional groups of the amino acid residues of RGD peptides. As the number of the functional groups in E[c(RGDfK)]{c[RGDf(4-I)K]} (**6**) is more than that of c[RGDf(4-I)K], it might significantly impede the stannylation reaction. Meanwhile, we expect that the reaction of the ^211^At-labeled dimer RGD peptide by the two-step method is possible because the ^211^At-labeled monomer RGD peptide was also synthesized by a similar two-step method [8]. However, the radiochemical yield of the ^211^At-labeled dimeric RGD peptide could prove to be too low by this method. Thus, modifying the labeling method might be necessary to improve the complication of the labeling procedure and the radiochemical yields of [^125/131^I]**6** and ^211^At-labeled dimer RGD peptides. For this purpose, a one-step radiolabeling method using a radiolabeling precursor without protecting groups would be required. To achieve the precursor synthesis, we will explore the direct stannylation reaction of a non-protected dimer RGD peptide by improving the metal-catalyzed stannylation reaction or investigating other stannylation reactions, such as photochemical stannylation reactions [15].

The binding affinity of **6** for α_v_β_3_ integrin (IC_50_: 1.2 ± 0.5 nM) was higher than that of c[RGDf(4-I)K] (IC_50_: 23.2 ± 17.2 nM) [11]. It was reported that IC_50_ values of dimeric RGD peptides in the α_v_β_3_ integrin competitive binding assay were an order lower than those of original monomeric RGD peptides [16]. Thus, the IC_50_ value of dimerizing peptide **6** was consistent with those described in previous studies.

This biodistribution study found that the accumulation of a dimeric RGD peptide, [^125^I]**6**, in the tumor tissue was higher than that of a monomeric RGD peptide, [^125^I]c[RGDf(4-I)K], as reflected in the results of the in vitro α_v_β_3_ integrin binding assay. Therefore, ^211^At-labeled dimeric RGD peptide could also accumulate highly in the tumor because the similar biodistribution of ^125^I- and ^211^At- labeled dimeric RGD peptides is expected [8]. However, the accumulation of [^125^I]**6** in the liver and intestines was higher than that of [^125^I]c[RGDf(4-I)K]; we suggest that the reason for this is the increased lipophilicity due to the dimerization of the RGD peptide. Notably, nearly all published studies of radiometal-labeled RGD peptides have reported that dimeric RGD peptides showed higher uptakes in the tumor and kidneys than did corresponding monomeric RGD peptides [9,16]. Although Log *p* values increased by dimerization in those reports, the Log *p* values were still much lower than those in this study. Thus, the renal excretion of the radiometal-labeled RGD peptides should not be changed. Dijkgraaf et al. proposed that the increased kidney uptake of multimeric RGD peptides was caused by two factors [16]: (1) the expression of β_3_ integrins on the endothelial cells of glomeruli vessels [17] and (2) the change in charge brought about by multimerization. Considering the results of this study and these previous reports, [^125^I]**6** could lead to higher accumulation in the kidneys by dimerization; however, the rate of hepatobiliary excretion increased due to increased lipophilicity. Thus, kidney accumulation of [^125^I]**6** did not change significantly compared with that of [^125^I]c[RGDf(4-I)K]. On the other hand, to further increase the tumor uptake, the introduction of a linker should be effective because it was reported that an appropriate distance between cyclic RGD peptides is important for multivalent effects [18].

In radionuclide therapy, a single administration of [^131^I]**6** (11.1 MBq) did not inhibit tumor growth in tumor-bearing mice (Figure 5). In a previous study, multiple administrations of the same dose of ^90^Y-labeled RGD peptide inhibited tumor growth in tumor-bearing mice; however, a single administration of ^90^Y-labeled RGD peptide did not affect tumor growth [19]. As no significant decrease in the body weight was observed in this study (Figure S2), a higher radiation dosage or multiple administrations may be appropriate and may further inhibit tumor growth. Meanwhile, it has also been reported that conjugation of a ^177^Lu-labeled RGD peptide with Evans Blue (EB) as an albumin-binding moiety positively affected its pharmacokinetics to elevate uptake and the residence time in the tumor, and it showed higher tumor growth inhibition than the ^177^Lu-labeled RGD peptide without EB [20]. Therefore, increased tumor accumulation of [^131^I]**6** by structural modification such as conjugation with an albumin binder may make it possible to inhibit tumor growth.

## 4. Materials and Methods

### 4.1. Materials

[^125^I]Sodium iodide (644 GBq/mg) and [^131^I]Sodium iodide (185 GBq/mg) were purchased from PerkinElmer (Waltham, MA, USA). Electrospray ionization mass spectra (ESI-MS) were obtained with a JEOL JMS-T100TD (JEOL Ltd., Tokyo, Japan). Purification and identification of peptides and labeled peptides were performed using an HPLC system (LC-20AD pump, SPD-20A UV detector at a wavelength of 220 nm, and CTO-20A column oven maintained at 40 °C; Shimadzu, Kyoto, Japan). Fmoc-Lys(Boc)-OH was purchased from Merck (Darmstadt, Germany). 2-Chlorotrityl chloride resin, Fmoc-Arg(Pbf)-OH, Fmoc-Asp(O*t*Bu)-OH, Fmoc-Gly-OH, Fmoc-d-Tyr(*t*Bu)-OH, Fmoc-d-Phe-OH, Fmoc-Lys(Alloc)-OH, Fmoc-Val-OH, and Fmoc-Glu-OAll were purchased from Watanabe Chemical Industries, Ltd. (Hiroshima, Japan). 1,4,7,10-Tetraazacyclododecane-1,4,7-tris(*t*-butyl acetate) (DOTA-tris) was purchased from Macrocyclics (Dallas, TX, USA). U-87 MG glioblastoma cells were purchased from DS Pharma Biomedical (Osaka, Japan). Fmoc-4-iodo-d-phenylalanine [Fmoc-d-Phe(4-I)] was synthesized according to a previous report [21]. *N*,*N*-Diisopropylethylamine (DIPEA) was purchased from Nacalai Tesque (Kyoto, Japan). 1,3-Diisopropylcarbodiimide (DIPCDI) and 1-hydroxybenzotriazole hydrate (HOBt) were purchased from Kokusan Chemical Co., Ltd. (Tokyo, Japan). 2-(1*H*-Benzotriazole-1-yl)-1,1,3,3-tetramethylaminium tetrafluoroborate (TBTU) was purchased from Chem Impex International, Inc. (Wood Dale, IL, USA). Other reagents were of reagent grade and used as received.

### 4.2. Synthesis of Reference Compounds and Radiolabeled Compounds

E[c(RGDfK)]{c[RGDf(4-I)K]} (**6**) and E[c(RGDfK)]{[^125/131^I]c[RGDf(4-I)K]} ([^125/131^I]**6**) were synthesized according to the procedure outlined in Scheme 1.

#### 4.2.1. Preparation of c[R(Pbf)GD(OtBu)fK] (**1**) and c[R(Pbf)GD(OtBu)f(4-I)K] (**2**)

Cyclic[Arg(Pbf)-Gly-Asp(O*t*Bu)-d-Phe-Lys] (**1**) and cyclic[Arg(Pbf)-Gly-Asp(O*t*Bu)-d-Phe(4-I)-Lys] (**2**) was synthesized manually using a standard Fmoc-based solid-phase methodology according to previous reports with slight modifications [8,22]. The crude peptide of **1** and **2** were purified by reversed phase (RP)-HPLC on Cosmosil 5C_18_-AR-II column (20 × 250 mm; Nacalai Tesque) at a flow rate of 12 mL/min with an isocratic mobile phase of 65% methanol in water with 0.1% trifluoroacetic acid (TFA). The solvents were removed by lyophilization to provide **1** (27.1 mg, 30%) and **2** (26.5 mg, 25%) as a white powder.

c[R(Pbf)GD(OtBu)fK] (**1**): MS (ESI^+^) calcd for C_44_H_65_N_9_O_10_S [M + H]^+^: *m*/*z* = 912.46 found: 912.79.

c[R(Pbf)GD(OtBu)f(4-I)K] (**2**): MS (ESI^+^) calcd for C_44_H_64_IN_9_O_10_S [M + H]^+^: *m*/*z* = 1038.36 found: 1038.54.

#### 4.2.2. Preparation of Fmoc-E{c[R(Pbf)GD(OtBu)fK]}-OAll (**3**)

Fmoc-Glu(OAll)-OH (29.6 mg, 72.4 µmol) was dissolved in 1 mL of DMF, and then TBTU (11.2 mg, 65.8 µmol) and DIPEA (23.0 µL, 132 µmol) were added to the solution. After stirring at room temperature for 1 h, compound **1** (20.0 mg, 22.0 µmol) was added to the reaction mixture. After further stirring for 1 h, the reaction mixture was purified by RP-HPLC with a Cosmocil 5C_18_-AR-II column (20 × 250 mm) at a flow rate of 12 mL/min with an isocratic mobile phase of 85% methanol in water with 0.1% TFA for 20 min. The solvent was removed by lyophilization to yield **3** (16.9 mg, 59%) as a white powder.

Fmoc-E{c[R(Pbf)GD(OtBu)fK]}-OAll (**3**): MS (ESI^+^) calcd for C_67_H_87_N_10_O_15_S [M + H]^+^: *m*/*z* = 1303.61; found, 1304.02.

#### 4.2.3. Preparation of Fmoc-E{c[R(Pbf)GD(OtBu)fK]}-OH (**4**)

Compound **3** (9.7 mg, 7.4 µmol) was dissolved in 1 mL of DMF, and then Pd(PPh_3_)_4_ (8.6 mg, 7.4 µmol) and phenylsilane (9.1 µL, 74 µmol) were added to the solution. After stirring at room temperature for 1 h, the reaction mixture was purified by RP-HPLC with a Cosmocil 5C_18_-AR-II column (20 × 250 mm) at a flow rate of 12 mL/min with a gradient mobile phase of 80% methanol in water with 0.1% TFA to 90% methanol in water with 0.1% TFA for 20 min. The solvent was removed by lyophilization to yield **4** (7.8 mg, 83%) as a white powder.

Fmoc-E{c[R(Pbf)GD(OtBu)fK]}-OH (**4**): MS (ESI^+^) calcd for C_64_H_83_N_10_O_15_S [M + H]^+^: *m*/*z* = 1263.58; found, 1263.95.

#### 4.2.4. Preparation of E{c[R(Pbf)GD(OtBu)fK)]{c[R(Pbf)GD(OtBu)f(4-I)K]} (**5**)

Compound **4** (7.8 mg, 6.2 µmol) was dissolved in 1 mL of DMF, and then TBTU (3.0 mg, 9.3 µmol) and DIPEA (3.3 µL, 19 µmol) were added to the solution. After stirring at room temperature for 1 h, compound **2** (9.6 mg, 9.3 µmol) was added to the reaction mixture. After 1 h stirring, solvent was removed by rotary evaporation then 1 mL of piperidine was added to the residue. After 1 h stirring at room temperature, purification was performed by RP-HPLC with a Cosmocil 5C_18_-AR-II column (20 × 250 mm) at a flow rate of 12 mL/min with an isocratic mobile phase of 85% methanol in water with 0.1% TFA for 20 min. The solvent was removed by lyophilization to yield **5** (6.9 mg, 54%) as a white powder.

E{c[R(Pbf)GD(OtBu)fK)]{c[R(Pbf)GD(OtBu)f(4-I)K]} (**5**): MS (ESI^+^) calcd for C_93_H_135_IN_19_O_22_S_2_ [M + H]^+^: *m*/*z* = 2060.85; found, 2061.94.

#### 4.2.5. Preparation of E[c(RGDfK)]{c[RGDf(4-I)K]}_2_ (**6**)

Compound **5** (3.0 mg, 1.5 µmol) was treated with a mixture of 95% TFA, 2.5% water, and 2.5% triisopropylsilane (TIS) for 2 h at room temperature. The crude peptide was purified by RP-HPLC on Cosmosil 5C_18_-AR-II column (10 × 250 mm) at a flow rate of 4 mL/min with a gradient mobile phase of 35% methanol in water with 0.1% TFA to 55% methanol in water with 0.1% TFA for 20 min. The solvent was removed by lyophilization to provide **6** (1.5 mg, 71%) as a white powder.

E[c(RGDfK)]{c[RGDf(4-I)K]}_2_ (**6**): MS (ESI^+^) calcd for C_59_H_87_IN_19_O_16_ [M + H]^+^: *m*/*z* = 1444.56; found, 1444.59.

#### 4.2.6. Preparation of E{c[R(Pbf)GD(OtBu)fK)]{c[R(Pbf)GD(OtBu)f(4-SnBu_3_)K]} (**7**)

Compound **5** (500 μg, 292 nmol) was dissolved in 500 µL of methanol. Bis(tributyltin) (23.3 µL, 46.6 µmol), tris(dibenzylideneacetone)dipalladium(0) (1.54 mg, 1.68 µmol), and DIPEA (4.8 µL, 28.0 µmol) were added to the solution of **5**. After stirring at 60 °C for 1 h, the reaction mixture was purified by RP-HPLC with a Cosmosil 5C_18_-AR-II column (10 × 250 mm) at a flow rate of 4 mL/min with a gradient mobile phase of 85% methanol in water with 0.1% TFA to 95% methanol with 0.1% TFA for 20 min. The solvent was removed by lyophilization to yield **7** as colorless oil.

E{c[R(Pbf)GD(OtBu)fK)]{c[R(Pbf)GD(OtBu)f(4-SnBu_3_)K]} (**7**) MS (ESI^+^) calcd for C_105_H_163_N_19_O_22_S_2_Sn [M + 2H]^2+^: *m*/*z* = 1112.53; found, 1112.98.

#### 4.2.7. Preparation of E[c(RGDfK)]{[^125^I]c[RGDf(4-I)K]}_2_ ([^125^I]**6**)

A small amount of **7** was dissolved in 5 μL of acetonitrile in the reaction vial. A measure of 10 μL of 1% acetic acid in acetonitrile, 3 μL of [^125^I]NaI (3.7 MBq) solution, and 15 μL of *N*-chlorosuccinimide (NCS) in acetonitrile (1 mg/mL) were added to the vial. After heating at 80 °C for 15 min, the reaction mixture was quenched with 15 μL of NaHSO_3_ solution (1 mg/mL). After evaporating the solvent using N_2_ gas, the residue was treated in 100 μL of a mixture of 95% TFA, 2.5% water, and 2.5% TIS for 90 min at room temperature, and then purified by RP-HPLC with a Cosmosil 5C_18_-AR-II column (4.6 × 150 mm) at a flow rate of 1 mL/min with a gradient mobile phase of 35% methanol in water with 0.1% TFA to 55% methanol in water with 0.1% TFA for 20 min. The radiochemical yield and the radiochemical purities of [^125^I]**6** were 38% and >96%, respectively.

#### 4.2.8. Preparation of E[c(RGDfK)]{[^131^I]c[RGDf(4-I)K]}_2_ ([^131^I]**6**)

[^131^I]**6** was synthesized in the same synthetic method as [^125^I]**6**, using [^131^I]NaI (185 MBq) instead of [^125^I]NaI. The radiochemical yields of [^131^I]**6** was 24%. The radiochemical purity of [^131^I]**6** was >92%.

### 4.3. α_v_β_3_ Integrin Binding Assay

Binding affinities of synthesized peptides E[c(RGDfK)]_2_ and **6** for α_v_β_3_ integrin were evaluated by competitive inhibition between the peptides and [^125^I]c[RGDy(3-I)V], which was prepared by Fmoc solid-phase synthesis and following ^125^I-labeling with chloramine-T method [23], to α_v_β_3_ integrin according to a previously reported procedure [24]. The peptides’ half maximal inhibitory concentration (IC_50_) values were calculated by curve fitting with nonlinear regression using GraphPad Prism 8.4.3 (GraphPad Software Inc., San Diego, CA, USA). Each data point is the average of four determinations, and IC_50_ values were expressed as mean ± standard deviation (SD) from three independent experiments.

### 4.4. Determination of the Partition Coefficient

The partition coefficient of [^125^I]**6** was measured as described previously [25]. The radioactivity in each phase was measured with an auto-well gamma counter. The partition coefficient was determined by calculating the ratio of 1-octanol to the buffer and was expressed as a common logarithm (log *P*).

### 4.5. In Vitro Stability

To evaluate the stability of [^125^I]**6** in PBS(−), 10 µL of each tracer (37 kBq) was added to 90 µL of PBS(−) (pH 7.4), and the solutions were incubated at 37 °C for 24 h. After incubation, the samples were drawn, and the radioactivity was analyzed by RP-HPLC.

### 4.6. Biodistribution of E[c(RGDfK)]{[^125^I]c[RGDf(4-I)K]}_2_ ([^125^I]***6***) in Tumor-Bearing Mice

Experiments with animals were conducted in strict accordance with the Guidelines for the Care and Use of Laboratory Animals of Kanazawa University. The experimental protocols were approved by the Committee on Animal Experimentation of Kanazawa University. The animals were housed with free access to food and water at 23 °C with a 12 h alternating light/dark schedule. We used a U-87 MG cell line because it was reported that the U-87 MG highly expresses α_v_β_3_ integrin [26]. U-87 MG cells were grown and inoculated subcutaneously into 4-week-old female BALB/c nude mice (15–19 g, Japan SLC, Inc., Hamamatsu, Japan) as previously reported [22]. Biodistribution experiments were performed approximately 14 days post-inoculation. A solution of [^125^I]**6** (37 kBq) was intravenously administered to groups of four mice. Mice were sacrificed at 1 and 4 h postinjection. Tissues of interest were removed and weighed. A neck containing thyroid was resected. The radioactivity counts of ^125^I was determined with an auto-well gamma counter (ARC-7010, Hitachi, Ltd., Tokyo, Japan) and corrected for background radiation and physical decay during counting. A window from 16 to 71 keV was used for counting ^125^I.

To investigate the effect of an excess amount of RGD peptide on biodistribution, U-87 MG tumor-bearing mice were intravenously administered 100 μL of a mixture solution of [^125^I]**6** (37 kBq) with c(RGDfK) peptide (0.2 mg/mouse). Mice (*n* = 4) were sacrificed at 1 h postinjection, and biodistribution experiments were conducted as described above.

### 4.7. Radionuclide Therapy

U-87 MG tumor-bearing mice (*n* = 4) as a treated group were intravenously administered 100 μL of [^131^I]**6** (11.1 MBq), and U-87 MG tumor-bearing mice (*n* = 3) as a control group were intravenously administered 100 μL of saline. The dose of [^131^I]**6** was unified with a previous therapeutic study with [^90^Y]Y-DOTA-c(RGDfK) (11.1 MBq) [19]. The tumor volume and body weight of mice were monitored 3–5 times weekly. The tumor size was measured with a slide caliper, and the tumor volume was calculated using the following formula: volume = 4/3 π (1/2 length × 1/2 width × 1/2 height).

### 4.8. Statistical Evaluation

Animal experiments were compared using unpaired Students’ *t*-test.

## 5. Conclusions

In this study, we synthesized an iodine-introduced dimer RGD peptide, E[c(RGDfK)]{c[RGDf(4-I)K]} (**6**), and an ^125/131^I-labeled dimer RGD peptide, E[c(RGDfK)]{[^125/131^I]c[RGDf(4-I)K]} ([^125/131^I]**6**) and evaluated them as a preliminary step toward the synthesis of an ^211^At-labeled dimer RGD peptide. The affinity of **6** for α_v_β_3_ integrin and the accumulation of [^125^I]**6** in tumor tissues of U-87 MG tumor-bearing mice improved compared to monomer RGD peptides. However, a single injection of [^131^I]**6** (11.1 MBq) did not inhibit tumor growth in tumor-bearing mice. This labeling method using the same precursor may be applicable to ^123^I-labeling for SPECT using E[c(RGDfK)]{[^123^I]c[RGDf(4-I)K]}_2_ ([^123^I]**6**) and ^211^At-labeling for TAT using an ^211^At-labeled dimer RGD peptide. The use of such radiolabeled peptides is expected to play a significant role in the future of clinical radiotheranostics.

## Supplementary Materials

The following are available online, Figure S1: The chromatogram of (a) [^125^I]**6** and (b) **6**, Figure S2: Body weight of U-87 MG tumor-bearing mice treated with [^131^I]**6** or with no treatment, Table S1: Biodistribution of radioactivity after intravenous injection of [^125^I]**6** in U-87 MG tumor bearing mice.

## Sample Availability

Samples of the compounds the synthesized compounds are available from the authors.

## References

[1] Kelkar S.S., Reineke T.M. (2011). Theranostics: Combining imaging and therapy. Bioconjug. Chem..

[2] Herrmann K., Schwaiger M., Lewis J.S., Solomon S.B., McNeil B.J., Baumann M., Gambhir S.S., Hricak H., Weissleder R. (2020). Radiotheranostics: A roadmap for future development. Lancet Oncol..

[3] Ogawa K. (2019). Development of diagnostic and therapeutic probes with controlled pharmacokinetics for use in radiotheranostics. Chem. Pharm. Bull..

[4] Mishiro K., Hanaoka H., Yamaguchi A., Ogawa K. (2019). Radiotheranostics with radiolanthanides: Design, development strategies, and medical applications. Coord. Chem. Rev..

[5] Parker C., Lewington V., Shore N., Kratochwil C., Levy M., Linden O., Noordzij W., Park J., Saad F., Targeted Alpha Therapy Working Group (2018). Targeted alpha therapy, an emerging class of cancer agents: A review. JAMA Oncol..

[6] Zalutsky M.R., Pruszynski M. (2011). Astatine-211: Production and availability. Curr. Radiopharm..

[7] Lindegren S., Albertsson P., Back T., Jensen H., Palm S., Aneheim E. (2020). Realizing Clinical trials with astatine-211: The chemistry infrastructure. Cancer Biother. Radiopharm..

[8] Ogawa K., Takeda T., Mishiro K., Toyoshima A., Shiba K., Yoshimura T., Shinohara A., Kinuya S., Odani A. (2019). Radiotheranostics coupled between an At-211-labeled RGD peptide and the corresponding radioiodine-labeled RGD peptide. ACS Omega.

[9] Janssen M., Oyen W.J., Massuger L.F., Frielink C., Dijkgraaf I., Edwards D.S., Radjopadhye M., Corstens F.H., Boerman O.C. (2002). Comparison of a monomeric and dimeric radiolabeled RGD-peptide for tumor targeting. Cancer Biother. Radiopharm..

[10] Ogawa K., Mizuno Y., Washiyama K., Shiba K., Takahashi N., Kozaka T., Watanabe S., Shinohara A., Odani A. (2015). Preparation and evaluation of an astatine-211-labeled sigma receptor ligand for alpha radionuclide therapy. Nucl. Med. Biol..

[11] Ogawa K., Echigo H., Mishiro K., Hirata S., Washiyama K., Kitamura Y., Takahashi K., Shiba K., Kinuya S. (2021). ^68^Ga- and ^211^At-Labeled RGD peptides for radiotheranostics with multiradionuclides. Mol. Pharm..

[12] Singh B., Fu C., Bhattacharya J. (2000). Vascular expression of the α_v_β_3_-integrin in lung and other organs. Am. J. Physiol. Lung Cell. Mol. Physiol..

[13] Ogawa K., Masuda R., Mishiro K., Wang M., Kozaka T., Shiba K., Kinuya S., Odani A. (2019). Syntheses and evaluation of a homologous series of aza-vesamicol as improved radioiodine-labeled probes for sigma-1 receptor imaging. Bioorg. Med. Chem..

[14] Effendi N., Ogawa K., Mishiro K., Takarada T., Yamada D., Kitamura Y., Shiba K., Maeda T., Odani A. (2017). Synthesis and evaluation of radioiodinated 1-{2-[5-(2-methoxyethoxy)-1H-benzo[d]imidazol-1-yl]quinolin-8-yl}piperidin-4-amin e derivatives for platelet-derived growth factor receptor beta (PDGFRbeta) imaging. Bioorg. Med. Chem..

[15] Sakamoto K., Nagashima Y., Wang C., Miyamoto K., Tanaka K., Uchiyama M. (2021). Illuminating stannylation. J. Am. Chem. Soc..

[16] Dijkgraaf I., Yim C.B., Franssen G.M., Schuit R.C., Luurtsema G., Liu S., Oyen W.J., Boerman O.C. (2011). PET imaging of alpha_v_beta_3_ integrin expression in tumours with ^68^Ga-labelled mono-, di- and tetrameric RGD peptides. Eur. J. Nucl. Med. Mol. Imaging.

[17] Wu Z., Li Z.B., Chen K., Cai W., He L., Chin F.T., Li F., Chen X. (2007). microPET of tumor integrin alpha_v_beta_3_ expression using ^18^F-labeled PEGylated tetrameric RGD peptide (^18^F-FPRGD4). J. Nucl. Med..

[18] Wang L., Shi J., Kim Y.S., Zhai S., Jia B., Zhao H., Liu Z., Wang F., Chen X., Liu S. (2009). Improving tumor-targeting capability and pharmacokinetics of ^99m^Tc-labeled cyclic RGD dimers with PEG_4_ linkers. Mol. Pharm..

[19] Yoshimoto M., Ogawa K., Washiyama K., Shikano N., Mori H., Amano R., Kawai K. (2008). α_v_β_3_ Integrin-targeting radionuclide therapy and imaging with monomeric RGD peptide. Int. J. Cancer.

[20] Zhao L., Chen H., Guo Z., Fu K., Yao L., Fu L., Guo W., Wen X., Jacobson O., Zhang X. (2020). Targeted radionuclide therapy in patient-derived xenografts using ^177^Lu-EB-RGD. Mol. Cancer Ther..

[21] Byk G., Cohen-Ohana M., Raichman D. (2006). Fast and versatile microwave-assisted intramolecular Heck reaction in peptide macrocyclization using microwave energy. Biopolymers.

[22] Ogawa K., Yu J., Ishizaki A., Yokokawa M., Kitamura M., Kitamura Y., Shiba K., Odani A. (2015). Radiogallium complex-conjugated bifunctional peptides for detecting primary cancer and bone metastases simultaneously. Bioconjug. Chem..

[23] Ogawa K., Takeda T., Yokokawa M., Yu J., Makino A., Kiyono Y., Shiba K., Kinuya S., Odani A. (2018). Comparison of radioiodine- or radiobromine-labeled RGD peptides between direct and indirect labeling methods. Chem. Pharm. Bull..

[24] Mizuno Y., Uehara T., Hanaoka H., Endo Y., Jen C.W., Arano Y. (2016). Purification-Free Method for Preparing Technetium-99m-labeled multivalent probes for enhanced in vivo imaging of saturable systems. J. Med. Chem..

[25] Ogawa K., Mukai T., Arano Y., Otaka A., Ueda M., Uehara T., Magata Y., Hashimoto K., Saji H. (2006). Rhenium-186-monoaminemonoamidedithiol-conjugated bisphosphonate derivatives for bone pain palliation. Nucl. Med. Biol..

[26] Zhou Y., Kim Y.S., Chakraborty S., Shi J., Gao H., Liu S. (2011). ^99m^Tc-labeled cyclic RGD peptides for noninvasive monitoring of tumor integrin α_v_β_3_ expression. Mol. Imaging.

