# Supplementary material for: Synthesis and Evaluation of a Dimeric RGD Peptide as a Preliminary Study for Radiotheranostics with Radiohalogens
Source: Molecules. 2021 Oct 10;26(20):6107. doi: 10.3390/molecules26206107 (PMC8539346; doi:10.3390/molecules26206107)
Supplement: Supplementary file 1 [file molecules-26-06107-s001.zip › molecules-1404176-supplementary.pdf]

Supporting Information

Synthesis and evaluation of a dimeric RGD peptide as a preliminary study for radiotheranostics with radiohalogens

Hiroaki Echigo [1], Kenji Mishiro [2], Takeshi Fuchigami [1], Kazuhiro Shiba [3], Seigo Kinuya [4], Kazuma Ogawa [1,2,*]

[1] *Graduate School of Medical Sciences, Kanazawa University, Kanazawa 920-1192, Japan*

[2] *Institute for Frontier Science Initiative, Kanazawa University, Kanazawa 920-1192, Japan*

[3] *Research Center for Experimental Modeling of Human Disease, Kanazawa University, Takara-machi 13-1, Kanazawa, Ishikawa 920-8640, Japan*

[4] *Department of Nuclear Medicine, Kanazawa University Hospital, Kanazawa University, Takara-machi 13-1, Kanazawa, Ishikawa 920-8641, Japan*

***Corresponding Author**

Institute for Frontier Science Initiative; Kanazawa University; Kakuma-machi, Kanazawa 920-1192; Japan

Telephone: 81-76-234-4460; Fax: 81-76-234-4459

E-mail: kogawa@p.kanazawa-u.ac.jp

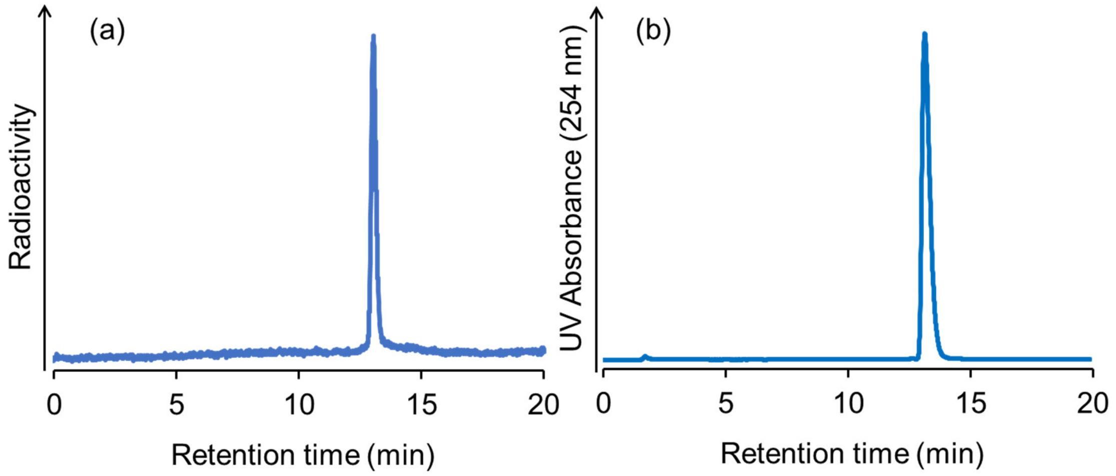

Figure S1. RP-HPLC chromatograms of (a) [^{125}I]**6** and (b) **6**. Condition: a flow rate was 1 mL/min with a gradient mobile phase of 35% methanol in water with 0.1% TFA to 55% methanol in water with 0.1% TFA for 20 min.

Table S1. Biodistribution of radioactivity after intravenous injection of [^{125}I]**6** in U-87 MG tumor bearing mice.

Tissues	1 h	4 h	Blocking (1 h)
Blood	0.68 (0.11)	0.40 (0.09)	0.38 (0.14) *
Liver	5.31 (0.46)	2.34 (0.46)	3.15 (1.17) *
Kidney	9.04 (0.89)	5.06 (0.90)	8.08 (1.91)
Small intestine	7.73 (2.08)	4.65 (1.06)	4.28 (1.59) *
Large intestine	1.71 (0.20)	16.8 (1.95)	0.26 (0.21) ***
Spleen	2.46 (0.53)	1.90 (0.28)	0.30 (0.13) ***
Pancreas	0.99 (0.11)	0.83 (0.12)	0.27 (0.23) **
Lung	2.01 (0.68)	1.13 (0.02)	0.45 (0.06) **
Heart	0.96 (0.30)	0.77 (0.08)	0.22 (0.06) **
Stomach ‡	0.98 (0.14)	0.56 (0.10)	0.28 (0.12) ***
Bone	1.56 (0.13)	1.10 (0.12)	0.30 (0.07) ***
Muscle	0.66 (0.08)	0.53 (0.03)	0.11 (0.04) ***
Brain	0.11 (0.04)	0.08 (0.02)	0.03 (0.01) ***
Neck‡	2.25 (0.14)	0.20 (0.05)	0.16 (0.03) ***
Tumor	5.87 (0.50)	4.12 (0.42)	0.45 (0.07) ***

Expressed as % injected dose per gram.

Each value represents the mean (SD) for four animals.

‡ Expressed as % injected dose.

Significance was determined by paired Student's t test.

* $p < 0.05$, ** $p < 0.01$, *** $p < 0.001$ vs control.

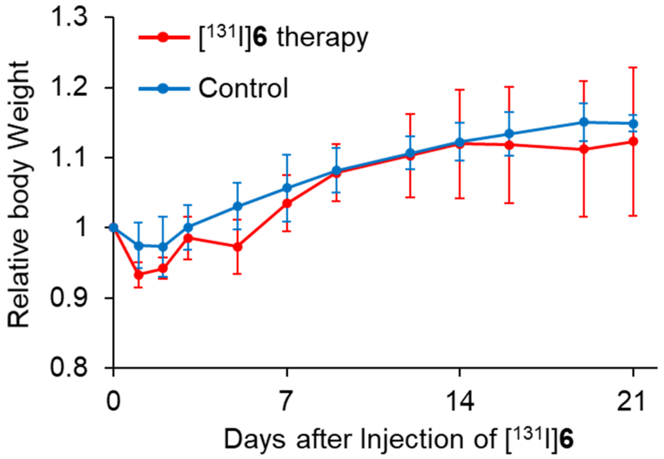

Figure S2. Body weight of U-87 MG tumor-bearing mice treated with $[^{131}I]$**6** or with no treatment. Data are expressed as relative value to initial body weight (mean ± SD).
